# Fitness costs associated with Vip3Aa resistance on various hosts in *Helicoverpa zea*

**DOI:** 10.1002/ps.70816

**Published:** 2026-04-15

**Authors:** Haley Kennedy, David L Kerns, Graham P Head, Fei Yang

**Affiliations:** ^1^ Department of Entomology Texas A&M University College Station TX USA; ^2^ Bayer Crop Science St. Louis MO USA; ^3^ Department of Entomology University of Minnesota Saint Paul MN USA

**Keywords:** *Bacillus thuringiensis*, fitness cost, *Helicoverpa zea*, resistance, Vip3A

## Abstract

**BACKGROUND:**

Transgenic crops containing *Bacillus thuringiensis* (Bt) proteins are crucial for managing major agricultural pests such as the corn earworm, *Helicoverpa zea* (Boddie). The fitness of Bt resistant insects on refuge crops plays an important role in determining the rate of resistance development. Previous research has investigated the fitness costs of Cry1 and Cry2 resistance in *H. zea*, but limited information is available about the fitness costs associated with Vip3Aa resistance in this pest. Here, we evaluated fitness costs of Vip3Aa‐resistant *H. zea* on three non‐Bt hosts: meridic diet, corn ears, and cotton leaves.

**RESULTS:**

On meridic diet, we detected recessive fitness costs resulting in lower survival and fecundity, and dominant fitness costs resulting in longer developmental time and reduced egg hatching rates. On corn ears, we observed recessive fitness costs resulting in lower pupal mass, and dominant fitness costs resulting in lower survival and fecundity, longer developmental time, and reduced egg hatching rates. On cotton leaves, we found recessive fitness costs resulting in lower female pupal mass, and dominant fitness costs resulting in lower adult survival and fecundity, and longer developmental time.

**CONCLUSION:**

Significant differences in survivorship, developmental time, pupal mass, and reproduction among the Vip3A‐resistant (RR), −susceptible (SS) and ‐heterozygous (R_♂_S_♀_ and R_♀_S_♂_) *H. zea* strains on the three hosts, revealed clear evidence of fitness costs associated with Vip3Aa resistance. These findings support the value of abundant non‐Bt refuges in limiting the evolution of *H. zea* resistance to Vip3Aa in the field. © 2026 The Author(s). *Pest Management Science* published by John Wiley & Sons Ltd on behalf of Society of Chemical Industry.

## Graphical abstract

Significant fitness costs associated with Vip3Aa resistance in *H. zea* were observed across three non‐Bt hosts. Abundant non‐Bt refuges could help limit the spread of Vip3Aa resistance in *H. zea* in the field.

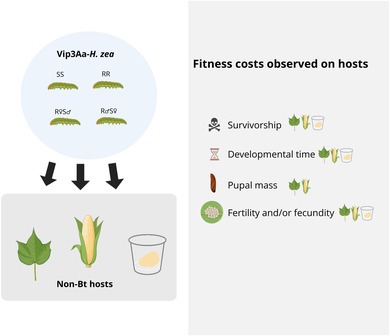

## 1 INTRODUCTION

*Helicoverpa zea* (Boddie), commonly known as the cotton bollworm or corn earworm, is one of the most economically important pests in the southern U.S., causing an estimated $74 million economic loss in cotton in 2022.^1^ The utilization of *Bacillus thuringiensis* (Bt) (Berliner) crops has played a vital role in the management of numerous lepidopteran species including *H. zea*. Field crops used for managing *H. zea* produce the Cry1s (Cry1A.105, Cry1Ab, Cry1Ac, and Cry1F), Cry2s (Cry2Ab and Cry2Ae), and Vip3Aa Bt proteins. Currently, Vip3Aa is incorporated into Bt corn and cotton products in the U.S., with Vip3Aa19 used in corn and Vip3Aa20 in cotton.^2^ However, the efficacy and durability of Bt technology is undermined by the evolution of resistance. Practical resistance to Cry1 and Cry2 proteins in *H. zea* has been widely reported in the southern U.S.^3^, ^4^, ^5^, ^6^, ^7^ As a result, Vip3Aa is currently the only effective Bt protein for *H. zea* management, which increases the risk of resistance.^7^, ^8^, ^9^, ^10^, ^11^ Although practical resistance to Vip3Aa in *H. zea* has not yet been reported, major Vip3Aa resistance alleles have been detected in the southern U.S. and a decline in susceptibility to Vip3Aa suggests early warning of Vip3Aa resistance in the field.^8^, ^11^, ^12^, ^13^

Insect resistant management (IRM) strategies such as the planting of refuges and use of pyramided crops that produce two or more Bt proteins have been widely used to delay the evolution of resistance to Bt crops. The refuge strategy works best when a Bt crop produces a high enough concentration of Bt protein to kill both homozygous susceptible (SS) and heterozygous (RS) insects, while nearby non‐Bt refuges produce synchronized SS moths to mate with the rare homozygous resistant (RR) moths emerging from the Bt crop. The offspring from these matings are expected to be RS and thus susceptible to the Bt crop, which helps slow the evolution of resistance to the Bt crops.^14^, ^15^ The inclusion of non‐Bt refuges with pyramided Bt crops is also necessary, as these refuges serve as a reservoir of susceptible insects to dilute resistance alleles in the population, thereby prolonging the effectiveness of pyramided Bt crops.^16^, ^17^, ^18^

Several factors can affect the success of Bt resistance management, such as non‐Bt refuge abundance, resistance allele frequency, dominance of resistance, cross‐resistance, redundant killing, incomplete resistance, and fitness costs associated with the resistance.^17^, ^19^, ^20^, ^21^, ^22^ Previous research suggests that abundant non‐Bt refuges can help delay Bt resistance development even if there are unfavorable conditions such as non‐recessive inheritance of resistance.^22^, ^23^, ^24^ In addition, fitness costs associated with Bt resistance can help slow the evolution of resistance.^18^, ^19^, ^20^, ^21^ Thus, ensuring the presence of abundant refuge is essential for successful Bt resistance management.^18^, ^19^, ^20^, ^25^ Fitness costs occur in the absence of Bt proteins, when the fitness of individuals carrying one or more resistance allele is lower than the fitness of susceptible individuals.^20^ Fitness costs have negative effects on life history traits such as survival, development, and fecundity.^20^, ^26^ Various environmental and ecological factors can influence the magnitude of fitness costs, including host plant species, host plant quality, natural enemies, allelochemicals, and intraspecific competition.^15^, ^26^, ^27^, ^28^, ^29^, ^30^, ^31^ For example, poor host plant quality increased fitness costs affecting developmental time in the diamondback moth, *Plutella xylostella*, (Karak and Keluang).^32^ Williams *et al*.^29^ and Carrière *et al*.^33^ provided evidence that adding gossypol, a polyphenolic aldehyde in cotton, to diet significantly increased the fitness cost in terms of larval developmental time in the pink bollworm, *Pectinophora gossypiella* (Saunders). Because the evolution of Bt resistance can be influenced by the relative fitness of insects carrying resistance alleles on non‐Bt hosts, magnifying fitness costs through refuge design can be an effective strategy to delay resistance. When fitness costs are recessive, RR insects exhibit reduced fitness compared to SS, while RS remains similar to SS insects. However, when fitness costs are non‐recessive or dominant, both RS and RR exhibit lower fitness than SS individuals, making these fitness costs more effective in slowing resistance development.^20^ Thus, refuges that enhance the expression of fitness costs could be particularly useful in sustainable resistance management.^19^, ^20^

Previous research has investigated the fitness costs of Cry1 and Cry2 resistance in *H. zea*, but relatively little is known about the fitness costs associated with Vip3Aa resistance in this pest.^21^, ^34^, ^35^, ^36^ Yang *et al*.^11^ established a Vip3Aa‐resistant strain of *H. zea* using an F_2_ screening method with populations collected from Texas in 2019. The availability of this Vip3Aa resistant *H. zea* strain in our laboratory provides a unique opportunity to examine how various non‐Bt hosts affect the fitness costs associated with Vip3Aa resistance, allowing us to quantify these fitness costs and develop strategies to increase the effectiveness of refuges to delay Vip3Aa resistance. In this study, we evaluated the fitness costs of Vip3Aa resistance using Vip3Aa‐SS, ‐RS and ‐RR *H. zea* and three non‐Bt hosts: meridic diet, corn and cotton. Dominant fitness costs resulting in lower survivorship and longer developmental times were observed across all three hosts, and recessive fitness costs resulting in lower pupal mass were detected on non‐Bt corn and cotton. These findings provide valuable information about the efficacy of current and potential refuge strategies for delaying field‐evolved resistance to the Vip3Aa in *H. zea*.

## 2 MATERIALS AND METHODS

### 2.1 Insect sources

In this study, a susceptible (SS) strain and a Vip3Aa‐resistant strain (RR) of *H. zea* were used as the original insect sources. The SS strain originated from Benzon Research Inc. (Carlisle, PA, U.S.) in 2017 and has demonstrated susceptibility to Cry1A.105, Cry1Ac, Cry2Ab2, and Vip3Aa39 in artificial diet bioassays.^11^ The RR strain was established via F_2_ screening with populations collected from Snook, Texas, U.S. in 2019, and exhibited >588.0‐fold resistance to Vip3Aa39 relative to the SS strain.^11^ The development of RR strain has been detailed in previous studies.^11^ The genetic basis of Vip3Aa resistance in the RR strain has been documented as autosomal, monogenic, and recessive.^37^ Both RR and SS strains were maintained in the laboratory on artificial diet, including liquid diet (Southland Product, Inc., Lake Village, AR, USA) and WARD'S Stonefly Heliothis diet (Rochester, NY, USA), as previously described.^38^ Approximately 450 individuals were used to maintain each generation of these strains. Prior to this study, to enhance genetic similarity between strains, the RR strain has been backcrossed with SS three times and then reselected with Vip3Aa39 protein using diet bioassays since its fully established in 2019. During each backcross, at least 50 male and 50 female insects were used for each reciprocal cross between RR and SS. If considering the initial establishment of RR strain, during which F₂ survivors were crossed with the SS strain,^11^ the RR had actually been crossed with SS a total of four times. The RR strain was selected on Vip3Aa39 at 5 μg cm^−2^ on diet‐overlay bioassays. The RR strain used in this study was derived immediately after the final round of backcrossing and reselection. In addition, two F_1_ heterozygous strains were produced from reciprocal crosses between the backcrossed reselected RR and SS, which were denoted as R♂S♀ and R♀S♂.

### 2.2 Vip3Aa39 protein

The Vip3Aa39 protein was offered by Dr. Juan Luis Jurat‐Fuentes from the University of Tennessee. Once received, the purified Vip3Aa39 protoxin was stored at −80 °C freezer. Vip3Aa39 was a solubilized protein at a concentration of 2.8 mg mL^−1^, which was produced by a recombinant *Escherichia coli* strain.^11^, ^39^

### 2.3 Host materials

Fitness costs associated with Vip3Aa resistance were investigated on three different food sources: non‐Bt meridic diet, non‐Bt corn ears, and non‐Bt cotton leaf tissues. The non‐Bt diet included liquid diet (Southland Product, Inc., Lake Village, AR, USA) and WARD'S Stonefly Heliothis diet (Rochester, NY, USA), which are very effective for rearing *H. zea* larvae.^38^ A non‐Bt cotton variety (DP 1822 XF) and a non‐Bt corn hybrid (DKC 6770 RR) were planted in separate fields with two different planting times at the Texas A&M University Field Laboratory in Snook, Texas. Each field was approximately one hectare. To prevent cross‐pollination and pollen contamination from other fields, the non‐ Bt corn field was isolated by >300 m from other corn fields at the same growth stage.^38^

### 2.4 Assessing fitness costs of Vip3Aa resistance in *H. zea*

To investigate the fitness of the four insect genotypes (SS, RR, R_♂_S_♀_, and R_♀_S_♂_) on the non‐Bt meridic diet, one neonate (<24 h) was placed in each cell of 128‐cell bioassay trays containing 800 μL of liquid diet (Southland Product, Inc., Lake Village, AR, USA). After 6 days, the surviving larvae were transferred into 30 mL plastic cups containing approximately 8 g of non‐Bt diet (WARD'S Stonefly Heliothis diet, Rochester, NY, USA). Once insects developed to pupal stage, pupa mass and sex were recorded and then transferred to new plastic cups and allowed to develop to adult stage. Insects were held in an insect rearing room maintained at 26 ± 1 °C, ~50% relative humidity (RH), and a photoperiod of 16:8 h (L:D). There were four replications for each insect genotype with 32 larvae in each replication (*n* = 32 x 4 = 128). Neonate‐to‐pupa survival, neonate‐to‐pupa developmental time, pupal mass and sex, neonate‐to‐adult survival, and neonate‐to‐adult developmental time were recorded daily.

To assess fitness costs on non‐Bt corn ears, larvae of the four genotypes were reared on excised non‐Bt corn ears. Selected ears along with husks and shanks were removed at the R1 stage (silking) from the field and brought to the laboratory. EnviroLogix QuickStix Kit (Portland, ME) was used to verify the absence of Bt proteins in kernels of different corn ears as described in previous studies.^38^, ^40^ After removing any naturally occurring insects, each ear was manually infested with two neonates on opposite sides of the ear. Infested ears were placed into 5.7 L sealed plastic containers with two pieces of paper towel underneath. The corn ears were replaced as needed during the experiment. Pupae were collected and placed into separate plastic cups until adult stage. The insects were maintained in the insect rearing room as previously mentioned.^38^ There were four replications for each insect genotype, and each replication contained 30 larvae (*n* = 30x4 = 120). The same set of developmental and survival parameters were recorded daily.

Fitness of the four insect genotypes on non‐Bt cotton leaves were assessed using excised, fully expanded leaves collected before first bloom. Leaves were collected from the top two nodes and plants were randomly sampled for providing leaves. The absence of Bt proteins in the cotton leaves was confirmed using a QuickStix™ kit as previously mentioned.^38^, ^40^ Three leaf pieces (≈3 cm long) were placed into a sterile Petri dish (100 × 15 mm) lined with moistened Whatman TM 90 mm (#1) filter paper as described in previous studies.^38^ Four neonates were placed on the leaf tissue and after 4 days, the surviving larvae were individually transferred into separate petri dishes to prevent cannibalism. Leaf tissues were changed every 1–2 days, and the filter paper was wetted and changed as needed. Pupae were removed from the petri dishes and placed into separate plastic cups until adult emergence. Insects were held in insect rearing room as previously mentioned. Each insect genotype was replicated four times, and each replication contained 32 larvae (*n* = 32 × 4 = 128). The same set of developmental and survival parameters were recorded daily.

Fecundity was also assessed for each insect genotype and host material. Sex of the insects was determined at the pupal stage. Five pairs of newly emerged (<24 h) male and female adults from the same genotype and host material were placed into a 0.9 L paper container (Choice Paper Company, Brooklyn, NY, USA) covered with cheesecloth as the egg substrate.^41^ The containers were placed in an insect rearing room and maintained at 26 ± 1 °C, ~50% RH with a photoperiod of 16:8 h (L:D) for mating and oviposition. For each insect genotype and host material, there were four replications. During the oviposition period, eggs were collected daily, washed from the cheesecloth with 5% bleach water and placed on filter paper to dry. Egg production per female (fecundity) was estimated from the average egg mass, daily total egg mass, and the number of female moths. To determine average egg mass, 200 eggs were counted and weighed. Total egg number was then estimated by dividing the total egg mass by the mass of 200 eggs and multiplying by 200. Eggs used for hatch rate assessment were collected from all four oviposition containers (replications) and eggs from each container were thoroughly mixed before sampling. Egg hatch rate (fertility) was assessed twice during the oviposition period. At each assessment, 200 eggs were randomly selected from the pooled eggs collected at that time, and hatch rate was calculated as the number of hatched neonates divided by 200.

### 2.5 Data analysis

Neonate‐to‐pupa developmental time, pupal mass, neonate‐to‐adult developmental time, and number of eggs per female were log(x + 1) transformed to improve assumptions of normality and homogeneity of variance. Neonate‐to‐pupa survival, neonate‐to‐adult survival, and egg hatch rate were arcsine (χ.05) transformed. The transformed data was analyzed using mixed models with insect genotype, food source, and their interaction as the fixed effects and replication as a random effect in SAS.^42^ Treatment means were compared using Tukey's HSD test at α = 0.05 level. Untransformed data are presented in the tables.

In addition, the magnitude of fitness costs was calculated to compare and quantify fitness cost components.^20^, ^25^ For fitness components positively associated with fitness (e.g., survival, pupal mass), the fitness ratio F_R/S_ was calculated as F_R_/F_S_, where F_R_ and F_S_ are the mean values of the fitness component for RR (or RS) and SS strains, respectively. For each of the fitness components, the percentage of fitness cost was calculated by (1‐F_R/S_) *100. Since developmental time is negatively associated with fitness, F_R/S_ was calculated as 1 − [(F_R_− F_S_)/F_S_] if the development time was greater for the RR (or RS) strain than SS, and 1 + [(F_S_− F_R_)/F_S_] if the development time was smaller for the RR (or RS) compared to SS.^25^

## 3 RESULTS

### 3.1 Survivorship

The effects of insect genotype, food source, and their interaction were all significant for both neonate‐to‐pupa and neonate‐to‐adult survivorship (Table 1). Across genotypes, survival from neonate‐to‐pupa and neonate‐to‐adult were similar on non‐Bt corn and non‐Bt cotton plant tissues but lower than on meridic diet (Table 2). On the meridic diet, neonate‐to‐pupa and neonate‐to‐adult survivorship was similar among SS, R_♂_S_♀_, and R_♀_S_♂_, with an average of 98.4% and 98.2%, respectively (Table 2). However, the RR genotype exhibited significantly lower survival compared to SS, with neonate‐to‐pupa and neonate‐to‐adult survival rates of 82.8% and 79.7%, respectively (Table 2). On non‐Bt corn, SS had the highest survival for both neonate‐to‐pupa and neonate‐to‐adult, which was higher than that of R_♂_S_♀_, R_♀_S_♂_, and RR. On non‐Bt cotton, neonate‐to‐pupa survivorship was similar for the insect genotypes, but significant differences occurred for neonate‐to‐adult survival (Table 2). SS had significantly higher neonate‐to‐adult survivorship compared to the other three insect genotypes (Table 2).

**Table 1:** Fixed effects for fitness cost parameters

Fitness cost parameter	Fixed effect	F‐value	DF	*P*‐value
Neonate‐to‐pupa survivorship	Insect genotype	20.25	3, 33	<0.0001
	Food source	609.09	2, 33	<0.0001
	Genotype × food source	8.31	6, 33	<0.0001
Neonate‐to‐adult survivorship	Insect genotype	30.19	3, 33	<0.0001
	Food source	770.99	2,33	<0.0001
	Genotype × food source	9.45	6, 33	<0.0001
Neonate‐to‐pupa developmental time	Insect genotype	90.92	3, 33	<0.0001
	Food source	2407.09	2, 33	<0.0001
	Genotype × food source	6.87	6, 33	<0.0001
Neonate‐to‐adult developmental time	Insect genotype	49.95	3, 33	<0.0001
	Food source	2659.97	2, 33	<0.0001
	Genotype × food source	25.63	6, 33	<0.0001
Female pupal mass	Insect genotype	32.60	3, 33	<0.0001
	Food source	2172.48	2, 33	<0.0001
	Genotype × food source	23.03	6, 33	<0.0001
Male pupal mass	Insect genotype	6.53	3, 33	0.0014
	Food source	1051.87	2, 33	<0.0001
	Genotype × food source	7.69	6, 33	<0.0001
Number of eggs per female	Insect genotype	14.01	3, 33	<0.0001
	Food source	63.64	2, 33	<0.0001
	Genotype × food source	2.80	6,33	0.0260
Egg hatch rate	Insect genotype	13.42	3, 33	<0.0001
	Food source	1.23	2, 33	0.3041
	Genotype × food source	5.13	6, 33	0.0008

**Table 2:** Survivorship and developmental data of SS, R_♂_S_♀_, R_♀_S_♂_, and RR genotypes of *H. zea* on non‐Bt meridic diet, non‐Bt corn, and non‐Bt cotton

Fitness cost parameter	Insect strain	Host plant					
		Diet		Corn		Cotton	
		*n* ^#^	Mean*	*n* ^#^	Mean*	*n* ^#^	Mean*
Neonate‐to‐pupa survival (%)	SS	126	98.4 ± 0.9 Aa	65	54.2 ± 1.6 Ca	65	50.8 ± 1.9 Ca
	R♂S♀	125	97.7 ± 0.8 Aa	54	44.5 ± 0.9 Cb	64	50.0 ± 1.3 Ca
	R♀S♂	127	99.2 ± 0.8 Aa	54	44.5 ± 0.9 Cb	62	48.4 ± 0.9 Ca
	RR	106	82.8 ± 2.9 Bb	49	40.8 ± 0.8 Cb	58	45.3 ± 2.0 Ca
Neonate‐to‐adult survival (%)	SS	125	97.7 ± 0.8 Aa	57	47.5 ± 2.1 Ca	59	46.1 ± 1.5 Ca
	R♂S♀	125	97.7 ± 0.8 Aa	46	38.3 ± 1.7 CDb	51	39.9 ± 0.9 CDb
	R♀S♂	127	99.2 ± 0.8 Aa	43	35.8 ± 1.6 CDbc	49	38.3 ± 0.8 CDbc
	RR	102	79.7 ± 3.3 Bb	36	30.0 ± 1.9 Dc	44	34.4 ± 1.3 CDc
Neonate‐to‐pupa developmental time (days)	SS	126	12.6 ± 0.2 Hc	65	16.1 ± 0.2 EFc	65	23.5 ± 0.2 Bb
	R♂S♀	125	13.9 ± 0.1 Gb	54	17.0 ± 0.1 DEb	64	25.1 ± 0.2 Aa
	R♀S♂	127	14.4 ± 0.3 Gb	54	17.5 ± 0.1 Db	62	24.7 ± 0.2 ABa
	RR	106	15.7 ± 0.1 Fa	49	19.1 ± 0.1 Ca	58	25.6 ± 0.4 Aa
Neonate‐to‐adult developmental time (days)	SS	125	22.5 ± 0.2 Fc	57	26.4 ± 0.03 Ca	59	33.2 ± 0.1 Bb
	R♂S♀	125	23.8 ± 0.1 Eb	46	25.7 ± 0.1 CDb	51	34.9 ± 0.1 Aa
	R♀S♂	127	25.0 ± 0.4 Db	43	25.6 ± 0.1 CDb	49	35.1 ± 0.1 Aa
	RR	102	26.2 ± 0.2 Ca	36	26.2 ± 0.3 Cab	44	35.5 ± 0.2 Aa
Male pupal mass (mg)	SS	63	293.7 ± 3.8 Ca	32	390.2 ± 7.3 Aa	33	217.9 ± 2.7 Da
	R♂S♀	62	307.4 ± 2.7 BCa	26	388.6 ± 3.4 Aa	31	214.1 ± 3.9 Da
	R♀S♂	68	305.2 ± 4.1 BCa	28	388.4 ± 3.4 Aa	30	207.7 ± 2.5 Da
	RR	53	312.3 ± 8.3 BCa	28	335.1 ± 4.2 Bb	31	205.7 ± 9.0 Da
Female pupal mass (mg)	SS	63	301.4 ± 3.6 Ca	33	397.7 ± 2.0 Aa	32	221.3 ± 2.0 Da
	R♂S♀	63	306.0 ± 5.6 Ca	28	395.7 ± 3.0 Aa	33	220.3 ± 5.2 Da
	R♀S♂	59	294.8 ± 6.4 BCa	26	383.2 ± 2.5 Aa	32	204.9 ± 2.3 Eb
	RR	53	313.0 ± 4.3 BCa	21	324.6 ± 3.9 Bb	27	203.7 ± 3.1 Eb

* Mean ± standard error (SE). Mean values followed by the same letter are not significantly different at α = 0.05 (Tukey's honestly significant difference (HSD) test). Comparisons were conducted within each fitness cost parameter. Uppercase letters indicate the difference among *H. zea* genotypes across all three hosts, whereas lowercase letters indicate the differences among *H. zea* genotypes within each individual host.

# n indicates sample size of insects used to calculate the corresponding fitness cost parameter.

### 3.2 Developmental time

The main effects of insect genotype and food source were significant for both neonate‐to‐pupa and neonate‐to‐adult developmental time (Table 1). The interaction between insect genotype and food source was also significant for the two developmental times. Overall, developmental time from neonate to pupa/adult for SS, R_♂_S_♀_, R_♀_S_♂_ and RR *H. zea* was shortest on meridic diet, followed by non‐Bt corn, and then non‐Bt cotton (Table 2). Within each food source, R_♂_S_♀_, R_♀_S_♂_, and RR consistently had longer neonate‐to‐pupa developmental time compared to SS (Table 2). Neonate‐to‐adult developmental time was also longer for the other three insect genotypes than SS when reared on meridic diet and non‐Bt cotton (Table 2). However, no delayed development from neonate‐to‐adult was observed for R_♂_S_♀_, R_♀_S_♂_, or RR on non‐Bt corn compared to SS (Table 2).

### 3.3 Pupal mass

The main effects of insect genotype, food source, and their interaction were all significant for both female and male pupal mass (Table 1). In general, pupal mass was highest on non‐Bt corn, followed by meridic diet, and lowest on non‐Bt cotton (Table 2). On meridic diet, male and female pupal mass were not different among SS, R_♂_S_♀_, R_♀_S_♂_, and RR, with an average mass of 304.7 mg for males and 303.8 mg for females (Table 2). On non‐Bt corn, pupal mass of SS, R_♂_S_♀_, and R_♀_S_♂_ were not different for either sex, whereas RR exhibited lower pupal mass compared to the other three genotypes. On non‐Bt cotton, male pupal mass did not differ among the four insect genotypes, with an average of 211.4 mg. However, female pupal mass was higher for SS and R_♂_S_♀_ than for R_♀_S_♂_ and RR (Table 2).

### 3.4 Reproductive data

The effects of insect genotype and food source on number of eggs per female produced were significant (Table 1). The interaction between insect genotype and food source was also significant. In general, egg production was higher on meridic diet and non‐Bt corn compared to non‐Bt cotton (Table 3). Across all food sources, SS females consistently produced the highest number of eggs compared to the other three insect genotypes. On meridic diet, SS females produced nearly 2.5 times more eggs than RR females. On non‐Bt corn and non‐Bt cotton, SS females produced an average of 785.8 and 324.9 eggs, respectively, which was more than that of R_♂_S_♀_, R_♀_S_♂_, and RR, averaging 532.2 eggs on non‐Bt corn and 242.3 eggs on non‐Bt cotton (Table 3). No differences in eggs production were observed among R_♂_S_♀_, R_♀_S_♂_ and RR on each food source, except between R_♂_S_♀_ and RR on non‐Bt corn (Table 3).

**Table 3:** Reproductive data of SS, R_♂_S_♀_, R_♀_S_♂_, and RR genotypes of *H. zea* on non‐Bt meridic diet, non‐Bt corn, and non‐Bt cotton

Fitness cost parameter	Insect strain	Host plant*		
		Diet	Corn	Cotton
Number of eggs per female produced	SS	916.3 ± 111.5 Aa	785.8 ± 15.5 ABa	324.9 ± 13.6 DEFa
	R♂S♀	429.1 ± 125.0 CDEab	582.3 ± 27.7 BCDb	267.5 ± 10.6 EFb
	R♀S♂	626.3 ± 102.0 ABCab	493.4 ± 17.3 ABCbc	227.7 ± 10.5 Fb
	RR	384.6 ± 51.9 CDEFb	521.0 ± 19.6 BCDc	231.7 ± 10.6 EFb
Egg hatch rate (%)	SS	68.8 ± 5.2 Aa	57.0 ± 2.8 ABa	50.4 ± 2.4 BCa
	R♂S♀	38.4 ± 4.6 Cb	54.1 ± 2.2 ABab	48.1 ± 0.5 BCa
	R♀S♂	38.2 ± 4.7 Cb	46.5 ± 1.2 BCb	44.8 ± 2.1 BCa
	RR	47.5 ± 7.5 BCb	45.6 ± 2.1 BCb	45.6 ± 1.6 BCa

* Mean ± standard error (SE). Mean values followed by the same letter are not significantly different at α = 0.05 (Tukey's honestly significant difference (HSD) test). Comparisons were conducted within each fitness cost parameter. Uppercase letters indicate the difference among *H. zea* genotypes across all three hosts, whereas lowercase letters indicate the differences among *H. zea* genotypes within each individual host.

Both the main effect of insect genotype and the interaction between insect genotype and food source influenced egg hatch rate (Table 1). In contrast, the main effect of food source was not significant. Significant differences in egg hatch rate among insect genotypes were observed on meridic diet and non‐Bt corn (Table 3). On meridic diet, R_♂_S_♀_, R_♀_S_♂_, and RR showed similar egg hatch rate (38.4, 38.2, and 47.5%, respectively), but all were lower than that of SS (68.8%). On non‐Bt corn, SS and R_♂_S_♀_ exhibited a higher egg hatch rate relative to R_♀_S_♂_ and RR. However, on non‐Bt cotton, egg hatch rate was similar among insect genotypes, ranging from 38.2 to 68.8% (Table 3).

### 3.5 Magnitude of fitness costs compared to the SS strain

Detailed calculations for the magnitude of fitness costs relative to the SS strain for each fitness parameter was presented in Table 4. In general, fitness costs in survivorship and pupal mass were greatest on non‐Bt corn ear among the three hosts. For example, the reduction in neonate‐to‐adult survival for RR and RS insects on corn ears was 36.9% and 19.4–24.6%, respectively, compared to 18.4% and − 1.5‐0.0% on meridic diet and 25.4% and 13.4–16.9% on cotton leaves. Similarly, the percentage of costs in pupal mass for RR on corn ears was 14.1–18.4%, whereas they ranged from −6.3‐0.0% on meridic diet and 5.6–8.0% on cotton leaves (Table 4). Fitness costs in terms of developmental time were greatest on meridic diet. Costs in neonate‐to‐pupa‐developmental time and neonate‐to‐adult developmental time for RR on meridic diet were 24.6 and 16.4%, respectively, compared with 18.6% and − 0.8% on corn ears and 8.9% and 6.9% on cotton leaves (Table 4). The magnitude of costs in number of eggs per female produced and egg hatch rate for RR and RS insects was also higher on meridic diet than on corn ears or cotton leaves (Table 4).

**Table 4:** The magnitude of the fitness cost relative to SS strain

Fitness cost parameter	Insect strain	% Fitness cost*		
		Diet	Corn	Cotton
Neonate‐to‐pupa survival	RR	15.9	24.7	10.8
	R♂S♀	0.7	17.9	1.6
	R♀S♂	−0.8	17.9	4.7
Neonate‐to‐adult survival	RR	18.4	36.9	25.4
	R♂S♀	0.0	19.4	13.4
	R♀S♂	−1.5	24.6	16.9
Neonate‐to‐pupa‐developmental time	RR	24.6	18.6	8.9
	R♂S♀	10.3	5.7	6.8
	R♀S♂	14.3	8.8	5.1
Neonate‐to‐adult developmental time	RR	16.4	−0.8	6.9
	R♂S♀	5.8	−2.7	5.1
	R♀S♂	11.1	−3.0	5.7
Male pupal mass	RR	−6.3	14.1	5.6
	R♂S♀	0.0	0.4	1.7
	R♀S♂	0.0	0.5	4.7
Female pupal mass	RR	0.0	18.4	8.0
	R♂S♀	0.0	0.5	0.5
	R♀S♂	2.2	3.6	7.4
Number of eggs produced per female	RR	58.0	33.7	28.7
	R♂S♀	53.2	25.9	17.7
	R♀S♂	31.7	37.2	29.9
Egg hatch rate	RR	30.9	20.0	9.5
	R♂S♀	44.2	5.0	4.6
	R♀S♂	44.8	18.4	11.1

* Indicates the percent of reduction in fitness parameters compared to SS strain. % fitness cost was calculated by (1‐F_R/S_) * 100. The F_R/S_ was calculated as F_R_/F_S_, where F_R_ and F_S_ are the mean values of the fitness component for the RR (or RS) and SS strains, respectively. For developmental time, F_R/S_ was calculated as 1 − [(F_R_− F_S_)/F_S_] if the development time was greater for the RR (or RS) strain than SS, and 1 + [(F_S_− F_R_)/F_S_] if the development time was smaller for the RR (or RS) compared to SS.

## 4 DISCUSSION

Previous studies have reported fitness costs associated with Vip3Aa resistance in other lepidopteran pests.^41^, ^43^, ^44^ However, little is known about the fitness costs associated with Vip3Aa resistance in *H. zea*.^21^, ^45^ In this study, we evaluated the fitness costs associated with Vip3Aa resistance in *H. zea* on artificial diet, non‐Bt corn ears and non‐Bt cotton leaves. Comparisons of life history traits among Vip3Aa resistant homozygous, reciprocal heterozygous and susceptible genotypes on different host materials revealed clear evidence of fitness costs associated with Vip3Aa resistance. On meridic diet, recessive fitness costs resulting in lower survival and fecundity, and dominant fitness costs resulting in longer developmental time and reduced egg hatching rates were detected. On corn ears, recessive fitness costs resulting in lower pupal mass, and dominant fitness costs resulting in lower survival and fecundity, longer developmental time, and reduced egg hatching rates were observed. On cotton leaves, we found recessive fitness costs resulting in lower female pupal mass, and dominant fitness costs resulting in lower adult survival and fecundity, and longer developmental time (Tables 2 and 3). It is important to note that before the onset of this study, the RR strain was backcrossed to the SS strain for a total of four times, therefore the genetic background of the RR strain was expected to be 93.75% similar to the SS strain. Although this approach minimized background genetic differences, complete genetic homogenization was not achieved, which could cause some overestimation of the dominance of fitness costs.^25^ Nevertheless, these findings provide strong evidence of fitness costs associated with Vip3Aa resistance in *H. zea*, supporting the value of abundant non‐Bt refuges in limiting the spread of Vip3Aa resistance alleles in *H. zea* in the field.

Two main approaches are commonly used to assess fitness costs associated with Bt resistance.^20^ The first involves comparing fitness components such as survival and developmental time between resistant and susceptible laboratory strains. The second evaluates the stability of resistance in heterogeneous laboratory strains containing both susceptible and resistant alleles when not exposed to Bt proteins. Recently, Carrière *et al*.^21^ used the second method to assess fitness costs of Vip3Aa resistance in *H. zea* by monitoring resistance changes in heterogeneous strains maintained on artificial diet without Vip3Aa exposure. They found a decrease in resistance in these heterogeneous strains, suggesting a fitness cost associated with Vip3Aa resistance in *H. zea*. In contrast, the present study used the first method to assess the fitness costs by comparing fitness components among homozygous, heterozygous and susceptible insects. Similar to Carrière *et al*.,^21^ we found fitness costs associated with Vip3Aa resistance in *H. zea*. For example, we observed dominant fitness costs for longer developmental time and reduced egg hatch rate, and recessive fitness cost for reduced number of eggs per female produced and lower survivorship (Tables 2 and 3). It is worth noting that the resistant *H. zea* strain in our study originated from a field collection, whereas the resistant strain examined by Carrière *et al*.^21^ resulted from continuous laboratory selections on Vip3Aa protein. Despite these differences in strain origin and selection history, both studies revealed fitness costs associated with Vip3Aa resistance in *H. zea*. Together, these findings suggest that fitness costs may be a general feature of Vip3Aa resistance in *H. zea*, providing important insights for assessing resistance risks in other Vip3Aa resistant *H. zea* populations.

In this study, we found that fitness costs associated with Vip3Aa resistance in *H. zea* were frequently non‐recessive. When resistance alleles are rare, heterozygotes are more common than homozygous resistant individuals in the field. Therefore, if fitness costs are non‐recessive, they can help reduce the frequency of resistance alleles in both heterozygotes and homozygous resistant populations and contribute more strongly to delaying resistance evolution. However, as mentioned above, the SS and RR strain did not have completely homogeneous genetic background and could perhaps have contributed in underestimating recessiveness of the costs. Carrière and Tabashnik reported a trend for studies using unrelated susceptible and resistant strains to overestimate the dominance of costs.^25^ Future studies using more genetically standardized strains would help confirm the degree of dominance of fitness costs associated with Vip3Aa resistance in *H. zea*. Nevertheless, the detection of frequent heterozygous disadvantages across multiple traits in our study suggests that non‐recessive fitness costs may play a critical role in slowing the evolution of Vip3Aa resistance in field populations of *H. zea*.

Host plant species can affect the magnitude of fitness costs associated with Bt resistance, therefore, refuges that magnify fitness costs are especially important for sustainable Bt resistance management.^28^ In the present study, fitness costs were greatest on non‐Bt corn in terms of survivorship and pupal mass (Table 4). For example, the reduction in neonate‐to‐adult survival for RR on corn was 36.9%, compared with 18.4% on meridic diet and 25.4% on cotton leaves. Similarly, the costs in pupal mass for RR on corn were 14.1–18.4%, compared with −6.3‐0.0% on meridic diet and 5.6–8.0% on cotton leaves. Regarding survivorship to pupal stage, no fitness cost was observed on non‐Bt cotton, recessive fitness cost was found on diet, but dominant fitness cost was evident on non‐Bt corn. Likewise, male pupal mass showed no fitness cost on diet or cotton but exhibited a recessive cost on non‐Bt corn. In a cross‐resistance study, Kennedy *et al*.^45^ reported evidence of dominant fitness costs in this Vip3Aa resistant *H. zea* population for neonate‐to‐pupa survivorship and developmental time, as well as recessive fitness costs for pupal mass on non‐Bt corn. In the current study, our comprehensive data confirm the findings of dominant fitness costs for neonate‐to‐pupa survivorship and developmental time in this Vip3Aa resistant *H. zea* population. Longer developmental time in the field could increase vulnerability to natural enemies and/or environmental stressors such as extreme weather or disease, which could further reduce survivorship.^46^ Similar to Kennedy *et al*.^45^ we also observed recessive fitness costs for pupal mass of both male and female on non‐Bt corn. However, compared to diet and non‐Bt cotton, pupal mass was generally highest on non‐Bt corn regardless of insect genotype, indicating that corn is the most suitable host for *H. zea*. Overall, our study suggests fitness costs associated with Vip3Aa resistance are present on non‐Bt corn ears, and thus synchronized planting of abundant non‐Bt corn refuges could maximize fitness costs and help slow the spread of Vip3Aa resistance in *H. zea*. However, maintaining abundant non‐Bt refuges or even increasing refuge size for *H. zea* is hampered by the lack of grower compliance with planting of structured non‐Bt refuges in the southern U.S, as well as the EPA's decision to abolish mandates for planting refuges of non‐Bt cotton in most states.^47^, ^48^, ^49^ In addition, the availability of refuge‐in‐bag (RIB) *versus* planting structured non‐Bt blocks may accelerate Bt resistance by increasing the dominance of resistance.^9^ As a result, the advantages of fitness costs associated with Bt resistance are unlikely to be fully realized for *H. zea* populations in the southern U.S. if the current low implementation of refuges continues.

Variation in fitness costs associated with Bt resistance on different host plant species can be affected by the nutritional quality and plant defense compounds of the host, which in turn affect the development and fecundity of insects.^50^, ^51^ Previous studies have shown that gossypol, a polyphenolic aldehyde in cotton, can affect larval development in Bt resistant strains of the pink bollworm, *Pectinophora gossypiella* (Saunders).^29^, ^33^ Williams *et al*.^29^ found that Cry1Ac‐resistant *P. gossypiella* exposed to gossypol in diet exhibited significantly lower larval mass and longer developmental time compared to susceptible insects not exposed to gossypol, thus increasing the fitness costs associated with Bt resistance. In this study, R_♂_S_♀_, R_♀_S_♂_, and RR exhibited a delay in developmental time to the pupal stage compared to SS, indicating fitness costs associated with developmental time. No measurable fitness costs of male pupal mass were detected; however, recessive fitness costs of female pupal mass were observed on non‐Bt cotton. Because gossypol is present in cotton leaves, it may have contributed to greater fitness costs in *H. zea*.^33^ In addition, because *H. zea* preferentially feed on cotton bolls, flowers, terminals, and squares, the use of cotton leaves in bioassays may have extended development time and reduced pupal mass, further amplifying the fitness costs on non‐Bt cotton.

In summary, this study provides a comprehensive evaluation of fitness costs associated with Vip3Aa resistance in *H. zea* on various hosts including artificial diet, non‐Bt corn, and non‐Bt cotton. Evidence of fitness costs, especially the non‐recessive costs, was very common in this study. Overall, data suggests that abundant non‐Bt refuges could help exploit these costs to manage Vip3Aa resistance in *H. zea*. Results from this study provide important information about the viability of current and potential refuge strategies for delaying field‐evolved resistance to Vip3Aa in *H. zea*.

## Data Availability

The data that support the findings of this study are available from the corresponding author upon reasonable request.
